# Do country-level environmental factors explain cross-national variation in adolescent physical activity? A multilevel study in 29 European countries

**DOI:** 10.1186/s12889-019-6908-9

**Published:** 2019-06-03

**Authors:** Dominic Weinberg, Gonneke W. J. M. Stevens, Jens Bucksch, Jo Inchley, Margaretha de Looze

**Affiliations:** 1 Department of Interdisciplinary Social Science, Universiteit Utrecht, Heidelberglaan 1, Postbus 80140, 3508 TC Utrecht, The Netherlands; 2 Fakultät III - Studiengang Prävention und Gesundheitsförderung, Pädagogische Hochschule Heidelberg, Postfach 10 42 40, 69032 Heidelberg, Germany; 3 Child and Adolescent Health Research Unit, School of Medicine, Medical & Biological Sciences, University of St Andrews, North Haugh, St Andrews, Fife KY16 9TF UK; 4 MRC/CSO Social and Public Health Sciences Unit, University of Glasgow, Top floor, 200 Renfield Street, Glasgow, G2 3AX UK

**Keywords:** Adolescence, Physical activity, Ecological theory, Environmental determinants, International comparison, Europe, Multilevel model, HBSC

## Abstract

**Background:**

Worldwide, roughly 80% of adolescents fail to meet World Health Organization (WHO) recommendations regarding physical activity, though there is substantial variation in adolescent physical activity prevalence across countries. This study explored whether country-level environmental differences explained cross-national variation in adolescent moderate- to vigorous-intensity physical activity (MVPA) and vigorous-intensity activity (VPA).

**Method:**

Using the data of 138,014 11- to 15-year-olds from 29 European countries in the 2013/2014 Health Behaviour in School-aged Children (HBSC) study, multilevel regression models examined the influence of four types of country-level environmental factors (physical, socio-cultural, economic, and political) on self-reported individual-level physical activity (MVPA and VPA).

**Results:**

The environmental variables explained 38% of country-level variance in MVPA and 81% of country-level variance in VPA. Lower annual average national temperature, higher community safety, lower average national household income and a weaker physical education policy were significantly associated with more MVPA. Greater urbanisation, lower annual average national temperature, higher adult physical activity and higher average national household income were significantly associated with more VPA.

**Conclusions:**

The findings showed that national differences in the physical, socio-cultural and economic environment were related to adolescent physical activity. They point to potential avenues for future research looking at interactions between individual and environmental factors.

**Electronic supplementary material:**

The online version of this article (10.1186/s12889-019-6908-9) contains supplementary material, which is available to authorized users.

## Background

Physical inactivity in adolescence is a critical public health issue [1]. The World Health Organization (WHO) recommends children and adolescents aged 5–17 accumulate at least 60 min a day of moderate- to vigorous-intensity physical activity (MVPA), and undertake vigorous-intensity physical activities (VPA) at least three times per week [2]. These recommendations are founded on evidence that moderate-intensity physical activity (performed at 3.0–5.9 times the intensity of rest, e.g. brisk walking, dance, and cycling to school) and VPA (more intense activity, e.g. running, soccer, and swimming laps) are important for short- and long-term health, including metabolic, musculoskeletal, cardiovascular and mental health [3, 4]. Furthermore, physical activity patterns typically track from childhood into adulthood [5], and the continuation of physical exercise throughout adulthood has been found to contribute to cognitive capacity and the prevention of dementia [6, 7].

However, levels of inactivity in adolescents and adults are high and estimated to cost $67.5 billion worldwide through health-care expenditure and productivity losses [8]. In 2010, 84% of girls and 78% of boys worldwide (aged 11–17) were insufficiently physically active [9], with older adolescents particularly unlikely to meet recommended targets; MVPA declines by as much as 7% per year during adolescence, though the trend in VPA is less clear [10, 11]. Despite recognition of the need for global action, there has been relatively little progress in increasing physical activity since 2010 [7, 12, 13].

Importantly, substantial variation in adolescent physical activity prevalence exists across and within countries [9, 14, 15], including within Europe [11, 13]. To illustrate, the 2013/2014 Health Behaviour in School-aged Children (HBSC) study found 41% of 11-year-olds in Finland, but only 13% of their counterparts in Italy, met WHO guidelines on daily MVPA. Correspondingly, 76% of Danish 15-year-olds, but only 30% of their Albanian counterparts, participated in VPA outside school for two or more hours per week [16].

International variation can be attributed to differences in the characteristics of individuals within a country, such as adolescent motivations for participation in physical activity [17]. Motivations, and therefore physical activity levels, may be explained by differences in country-level environmental factors [18]. Ecological models emphasise the importance of the environment as a context for physical activity [19]. Empirical research supports this theoretical approach and findings indicate that physical activity behaviour is affected by environmental influences across recreational, transport, household and occupational domains [20]; national differences in these environments might contribute to cross-national variation in adolescent physical activity [21]. The ecological Analysis Grid for Environments Linked to Obesity (ANGELO) framework provides a useful tool for distinguishing four types of environmental factors relevant to physical activity: physical, socio-cultural, economic and political [22]. The framework has been used to understand how cross-national environmental variation facilitates or hinders adult physical activity [23, 24], but has not yet been applied in multilevel analysis with adolescents.

Among adolescents, studies have identified several different characteristics of the *physical environment* to be important for physical activity. Urbanisation contributes to land-use mix (i.e. where housing is near commercial and institutional destinations, raising neighbourhood walkability [25]); systematic reviews have concluded that facilities for physical activity (e.g. gyms), population density and land-use mix are positive correlates of physical activity in adolescents [26, 27]. Temperature is another important aspect of the physical environment, with extreme conditions making physical activity less appealing [28]. Seasonal effects, with adolescents in many countries more physically active during warmer months, have been widely found, but cross-national analysis shows a more complex relationship between national temperature and physical activity, with less activity in hot countries [29].

The effect of the *socio-cultural environment* (i.e. attitudes, beliefs and values about physical activity) is better understood at the micro (e.g. home and school) level than at the macro (regional or country) level [30]. Adult physical activity may be associated with adolescent physical activity because adolescents are potentially influenced by the norms of others. However, empirical research on the effect of norms at the national level is inconsistent [31, 32]. Subjective assessments of community safety may also be an important aspect of the socio-cultural environment, for example due to the effect of perceptions on appropriateness of children spending time outdoors and in active transportation [33, 34]. For older adolescents the evidence of the effect of safety is more mixed [26].

The national *economic environment* (i.e. wealth and its distribution in the country of residence) has complex cumulative effects on adolescent health [35]. These effects include its impact on material resources that support physical activity (e.g. facilities) and the social consequences of inequality, including increased stress and social disorder [36]. Public health models typically examine national measures of income inequality and income together [37] and international research has found more adolescent MVPA in countries with higher national income and lower income inequality [38]. This effect was found even after controlling for an individual-level measure of wealth inequality, given consistent findings that children from more affluent families are more physically active [11, 13].

Finally, the role of *the political environment* (i.e. legislative and regulatory actions) has been understudied in physical activity research [20], despite its potential contribution to explaining cross-national differences in adolescent physical activity. One systematic review found evidence that policies promoting physical education in school and active transport (i.e. walking or cycling to school) increased physical activity in adolescents [39].

Research findings therefore suggest that all four types of environmental factors may be relevant for understanding international differences in adolescent physical activity [23, 38]. However, there is a scarcity of internationally comparative research testing the relative importance of the different types of environmental factors for adolescent physical activity. It is important to examine the effects of the environment on both MVPA and VPA, given their separate contribution to adolescent health [4], and the need to understand behaviour-specific environmental correlates [40, 41].

To address these gaps in the literature, this study includes eight national environmental factors (two from each of the four types in the ANGELO framework) to investigate whether cross-national differences in these factors explain international variation in both adolescent MVPA and adolescent VPA. We hypothesised that national differences in these environmental factors would explain a substantive amount of the international variation in adolescent physical activity. Based on the evidence discussed above, we expected to find higher levels of MVPA and VPA among adolescents living in countries with lower national temperatures, greater urbanisation, higher adult physical activity, higher community safety, higher national income, lower income inequality, and physical education and transport policies that supported physical activity.

## Methods

### Participants

Individual-level data were obtained from the 2013/2014 Health Behaviour in School-aged Children (HBSC) study [16]. This WHO collaborative cross-national study has investigated the health behaviours, well-being and social environment of adolescents since 1983 by self-report. The 2013/2014 survey collected data from roughly 4500 adolescents in each of 42 countries in Europe, North America, and Israel. Each country or region used cluster sampling, selecting schools and classes to get a representative sample of boys and girls aged 11, 13 and 15. Data collection took place between September 2013 and January 2015, with the majority of countries (22 out of 29) conducting fieldwork between January and June 2014 (p. 239, [16]). All participating countries adhere to a standard international protocol to ensure consistency of measures, sampling and implementation procedures [42]. Appropriate ethical approval for the survey was gained at national level. Adolescents and their parents were given age-appropriate information about the study’s approach to confidentiality and anonymity. Participation was voluntary, and passive consent was sought from school administrators, parents and adolescents, according to local human subject requirements.

The present analyses were based on adolescents from 29 of the 42 HBSC countries and regions (*N* = 149,660); 13 countries were excluded due to missing data on macro-level variables. Adolescents with a missing value for school (*n* = 68) or a missing value for at least one of the individual-level measures described below (*n* = 11,578) were also excluded from the analyses. Adolescents lived in European countries with ‘very high’ scores on the Human Development Index (HDI > .8), except Bulgaria (HDI = .794) [43]. Data for England, Wales and Scotland, and the French and Flemish regions of Belgium, were analysed separately. Whenever macro-data were not available for these administrative areas, data for the United Kingdom and Belgium were used. (Further mentions in text refer to all macro-level areas as countries.)

### Measures

Individual-level data on age, gender, school, and physical activity (MVPA and VPA) were obtained from the HBSC study. Country-level data on environmental factors were obtained from internationally recognised online sources used by other researchers.

#### Physical activity

The HBSC questionnaire defines MVPA as “any activity that increases your heart rate and makes you get out of breath some of the time”, and gives examples of such activity, e.g. biking and dancing (examples could be country-specific). *MVPA* was assessed with the question: “Over the past 7 days, on how many days were you physically active for a total of at least 60 minutes per day? Please add up all the time you spent in physical activity each day.” Responses ranged from 0 to 7 days and this measure was treated as a continuous variable. *VPA* was assessed with the question: “Outside school hours: how many hours a week do you usually exercise in your free time so much that you get out of breath or sweat?” Answers were recoded to form a continuous scale, ranging from 0 (*none*) to 7.5 (*about 7 h or more*). Both items have been found to have reasonable validity and moderate reliability [44].

#### Physical environment

*Annual average national temperature* data were obtained from Weatherbase for 2017 [45]. This website collects international weather data from public domain sources and was used by Lang and colleagues [46]. National temperature was computed by averaging the annual temperature across major cities in each country using at least 10 years of data. We used nationally representative samples from highly urbanised countries, and as such, the temperature of major cities as assessed with the Weatherbase data was expected to approximate the experience of most adolescents. *Urbanisation* data were obtained from the World Bank DataBank for 2014 [47]. The data, collected and smoothed by United Nations Population Division, contains national estimates of urban population (% of total) for 2014. Urban population refers to the percentage of people living in urban areas as defined by national statistical offices; Eurostat defines urban areas as clusters of contiguous grid cells of 1km^2^ with a density of at least 300 inhabitants per km^2^ and a minimum population of 5000 [48].

#### Socio-cultural environment

*Adult physical activity* data were obtained from the Global Health Observatory data repository for 2010 [49]. The repository contains national data on insufficient physical activity prevalence in adults. Insufficient physical activity was defined as the percentage of adults aged 18 and over doing fewer than 150 min of moderate physical activity per week, and was estimated from population-based surveys, sometimes adjusted by WHO (e.g. due to unrepresentative survey coverage) to enable comparison among countries. Data were recoded to assess the percentage of adults engaged in sufficient physical activity. *Safety* data were obtained from the European Social Survey [50]. The cross-national survey asked a representative sample of people aged 15 and over in each country whether they felt safe walking alone in the local area after dark; answer categories were “very safe”, “safe”, “unsafe”, “very unsafe”, and “don’t know”. Post-stratification weights were used to adjust for sampling error, non-response bias and different selection probabilities. For this study, we calculated the percentage of respondents who reported feeling safe or very safe in the most recent survey for which data were available in each country (2008, 2010, 2012 or 2014).

#### Economic environment

*Average national household income* data were obtained from Eurostat for 2014 [51]. The Eurostat database contains national data on adjusted gross disposable income of households per capita in purchasing power standard (an artificial common currency). Adjusted disposable income takes into account transfers in-kind, such as government provided education and health, and has been proposed to be a better indicator of the material well-being of citizens than gross domestic product per capita (GDP) [52]. *Income inequality* data were obtained from Eurostat for 2014 [53]. The European Union Statistics on Income and Living Conditions (EU-SILC) instrument provides estimates of cross-national standardised Gini indices of equivalised disposable income inequality. The Gini index theoretically ranges from 0 (everyone having equal income) to 100 (one person having all the income).

#### Political environment

*Physical education policy* data were obtained from the Eurydice network for 2011/12 [54]. The network collected data on the required minimum annual taught time (in hours) for physical education as a compulsory subject in full-time education for children aged 11–15. In Belgium (Flanders), Netherlands, England and Wales, despite a requirement for schools to deliver physical education, there was no specified minimum time. Instead, schools were responsible for deciding the number of hours, so these countries were coded as 1 h to approximate the minimum time required (i.e. just greater than 0). *Transport policy* data were obtained from the WHO Regional Office for Europe for 2009 [55]. National experts completed questionnaires and recorded the existence of national or subnational schemes promoting active travel to school. Responses were coded as 0 for countries with no such policies and 1 for countries with policies [56].

### Data analysis

Statistical analyses were conducted with Mplus version 7 using the maximum likelihood estimator with robust standard errors [57]. The associations between individual-level physical activity outcomes and country-level variables were tested by fitting three-level linear regression models, which considered individuals to be clustered within schools (*n* = 5109) and within countries (*n* = 29). The individual- and country-level determinants were added to the models using a stepwise approach. Model 1 included individual-level variables only, to assess whether significant variation between countries in physical activity outcomes existed after adjusting for age and gender. The country-level intraclass correlation (ICC) measured the proportion of variance in physical activity attributable to country-level variation. Model 2 included individual-level variables and all country-level variables. We included only significant variables in the final model, Model 3. Due to large number of predictors and the possibility of correlations between them and suppression effects, we adopted a less conservative α-level of .1 as a screening criterion for entry into the final model, Model 3, to ensure that all potentially relevant variables were considered in the final model [58, 59]. For testing the significance of variables in the final model, α was .05. The model building sequence was followed for MVPA and VPA separately.

## Results

Table 1 shows the descriptive characteristics of the sample, which reveals substantial variance across countries for all outcome and country-level variables. One hundred thirty-eight thousand fourteen adolescents were included in the analysis (mean age 13.6 years old, 51% female). MVPA averages in 15-year-olds ranged from 3.20 days per week (Italy) to 4.22 days per week (Finland) and VPA averages in 11-year-olds ranged from 1.78 h per week (Portugal) to 3.90 h per week (Netherlands). Correlations between the country-level variables are shown in Table 2. National income positively correlated with urbanisation and safety, and negatively correlated with adult physical activity and income inequality. Income inequality positively correlated with national temperature and negatively correlated with safety (and national income).

**Table 1:** Descriptive characteristics of the individual- and country–level sample (*N* = 138,014)

Sample			Individual-Level Characteristics								Country-Level Characteristics							
					11-year-olds		13-year-olds		15-year-olds		Physical		Socio-cultural		Economic		Political	
Country	*n*	*n* schools	Mean age	Girls (%)	MVPA	VPA	MVPA	VPA	MVPA	VPA	Temp. (°C)	Urban (%)	Adult PA (%)	Safety (%)	Ntl. inc.	Inc. ineq.	PE policy	Trans. policy
Austria	3,351	204	13.40	53.06	4.87	3.12	4.53	3.08	3.45	2.87	7.0	65.9	76.2	84.5	25,927	27.6	90.0	1
Belgium (Flemish)	3,954	58	13.64	46.13	4.17	3.29	3.98	3.06	3.67	3.32	9.0	97.8	66.8	79.9	23,778	25.9	1.0	0
Belgium (French)	5,795	190	13.47	50.27	4.20	2.83	3.97	2.82	3.72	2.88	9.0	97.8	66.8	79.9	23,778	25.9	76.0	0
Bulgaria	4,419	160	13.73	47.61	4.75	2.07	4.39	2.04	4.06	1.99	9.6	73.6	79.0	58.3	9,236	35.4	57.5	0
Croatia	5,138	223	13.65	50.12	4.94	1.87	4.60	2.20	4.01	2.08	12.5	58.7	83.8	87.3	12,496	30.2	53.0	1
Czech Republic	4,847	93	13.46	52.36	4.62	1.99	4.47	2.48	3.99	2.52	6.8	73.0	76.2	74.0	16,086	25.1	59.0	0
Denmark	3,510	48	13.78	53.79	3.87	3.03	3.83	3.67	3.67	3.71	7.5	87.5	75.7	88.2	22,458	27.7	75.0	1
England	4,416	50	13.62	49.68	4.63	2.26	4.29	2.38	3.83	2.45	9.7	82.3	62.7	76.2	22,297	31.6	1.0	1
Estonia	3,985	90	13.76	50.09	4.21	2.05	3.98	2.44	3.77	2.61	5.5	67.6	88.1	69.4	13,882	35.6	61.3	1
Finland	5,677	359	13.77	51.51	5.46	3.35	4.78	3.42	4.22	3.48	2.7	84.1	76.5	91.3	23,282	25.6	57.0	1
France	5,180	240	13.48	49.46	3.90	2.40	3.68	2.55	3.31	2.43	11.2	79.3	76.2	73.4	24,113	29.2	117.0	1
Germany	5,500	172	13.50	49.13	4.18	2.91	3.92	3.03	3.58	3.01	7.8	75.1	78.9	77.9	27,070	30.7	60.8	1
Greece	3,995	243	13.65	50.31	3.99	2.63	3.88	2.65	3.30	2.37	16.9	77.7	87.1	52.9	15,249	34.5	58.5	0
Hungary	3,745	255	13.41	50.49	4.60	2.15	4.08	2.30	3.86	2.43	10.0	70.8	81.9	71.3	13,178	28.6	69.0	0
Ireland	3,556	163	13.71	61.30	5.27	2.59	4.62	2.56	3.92	2.48	9.6	63.0	64.9	77.7	19,403	31.1	43.0	0
Italy	3,906	177	13.67	50.03	3.51	2.07	3.45	2.40	3.20	2.49	13.5	68.8	66.8	70.1	20,813	32.4	66.0	0
Latvia	5,470	131	13.59	52.49	4.28	2.04	4.05	2.21	3.95	2.44	6.0	67.4	78.0	49.5	11,979	35.5	46.0	0
Lithuania	5,576	131	13.58	49.52	4.35	2.22	4.17	2.79	4.00	3.00	6.2	66.5	81.6	57.9	15,432	35.0	60.8	0
Netherlands	4,160	145	13.50	51.18	4.47	3.90	4.41	3.83	4.15	3.61	9.3	89.9	84.5	85.3	22,729	26.2	1.0	1
Norway	2,595	134	13.38	52.41	4.74	3.42	4.18	3.71	3.84	3.84	4.3	80.2	74.2	90.0	27,745	23.5	72.1	1
Poland	4,420	196	13.58	50.34	4.97	2.45	4.42	2.19	3.87	2.09	6.9	60.6	81.3	84.7	14,274	30.8	100.4	0
Portugal	4,704	35	13.51	52.38	4.11	1.78	3.75	1.91	3.41	1.93	15.7	62.9	65.1	81.9	16,342	34.5	79.3	0
Romania	3,886	150	13.21	52.88	4.36	1.94	3.92	1.96	3.22	1.85	8.4	54.4	74.7	66.8	12,376	35.0	44.5	1
Scotland	5,450	266	13.68	50.66	4.70	3.17	4.23	2.99	3.83	2.80	8.1	82.3	62.7	76.2	22,297	31.6	76.0	1
Slovakia	5,211	130	13.48	50.95	4.70	2.40	4.43	2.20	4.11	2.22	6.2	53.8	82.2	70.2	15,444	26.1	56.0	1
Slovenia	4,753	262	13.63	51.40	4.61	2.32	4.21	2.46	3.58	2.29	7.7	49.7	78.7	93.3	16,360	25.0	76.8	0
Spain	9,032	336	13.61	51.64	4.91	2.19	4.55	2.46	4.21	2.56	15.5	79.4	69.5	82.0	18,245	34.7	39.5	0
Sweden	7,053	386	13.63	50.69	4.31	2.85	3.93	3.00	3.84	3.24	4.7	85.7	71.3	85.6	23,481	25.4	55.6	0
Wales	4,730	82	13.71	49.53	4.24	2.16	4.06	2.50	3.55	2.45	9.9	82.3	62.7	76.2	22,297	31.6	1.0	1
Mean	4,759	176.17	13.58	50.97	4.49	2.52	4.18	2.63	3.79	2.67	8.9	73.7	75.0	76.3	19,036	30.1	57.0	0.48

*MVPA* Moderate- to vigorous-intensity physical activity (days/week), *VPA* Vigorous-intensity physical activity (hours/week), *Temp*. National temperature, *Adult PA* Adult physical activity, *Ntl. inc.* National income, *Inc. ineq.* Income inequality, *PE* Physical Education, *Trans.* Transport

**Table 2:** Correlations between country-level variables

	Variables	2	3	4	5	6	7	8 ^a^
Physical	1. National temperature	−.03	−.17	−.19	−.20	.45*	−.07	−.19
	2. Urbanisation		−.34	.16	.61**	−.30	−.32	.12
Socio-cultural	3. Adult physical activity			−.22	−.44*	.03	.24	.05
	4. Safety				.54**	−.67**	.08	.20
Economic	5. National income					−.55**	−.04	.33
	6. Income inequality						−.06	−.10
Political	7. PE policy							−.05
	8. Transport policy							

^a^Transport policy correlation coefficients are Spearman’s (other correlations are Pearson). *PE* Physical Education, * *p* < .05, ** *p* < .01

Table 3 displays results of MVPA analyses, with models showing that both gender and age were significant individual-level correlates of MVPA, with less MVPA among girls and older adolescents. Model 1 shows significant school- and country-level variance. The residual ICC – i.e. variance attributable to differences at a higher level after controlling for age and gender – was 3.4% at school-level and 2.6% at country-level. Model 2 included all country-level variables, and national temperature, safety, national income, and physical education policy met the criterion (α-level of .1) for inclusion in the final model. Model 3 shows that when these variables were included in a regression together, lower national temperature, higher safety, lower national income, and a weaker physical education policy were significantly associated (at an α-level of .05) with more MVPA. The country-level variables in Model 3 explained 38% of the total country-level variance.

**Table 3:** Multilevel models for moderate- to- vigorous-intensity physical activity with unstandardised and standardised fixed effects at individual- and country-level (*N* = 138,014)

		Model 1			Model 2			Model 3		
		*b* (SE)	*p*	*β*	*b* (SE)	*p*	*β*	*b* (SE)	*p*	*β*
Fixed effects (individual -level)	Intercept	6.096 (0.196)	< .001		5.649 (1.505)	< .001		6.081 (0.357)	< .001	
	Gender ^a^	**0.619 (0.037)**	< .001	.16	**0.619 (0.037)**	< .001	.16	**0.619 (0.037)**	< .001	.16
	Age	**−0.167 (0.012)**	< .001	−.14	**−0.167 (0.012)**	< .001	−.14	**−0.167 (0.012)**	< .001	−.14
Fixed effects (country-level)	Ntl. temperature				**−0.039 (0.015)**	.010	−.39	**−0.036 (0.017)**	.032	−.37
	Urbanisation				0.001 (0.007)	.923	.03			
	Adult PA				0.001 (0.007)	.867	.03			
	Safety				**0.016 (0.007)**	.017	.54	**0.014 (0.004)**	.001	.49
	Ntl. income				−0.029 (0.016)	.072	−.46	**−0.031 (0.009)**	.001	−.49
	Income inequality				0.006 (0.019)	.732	.08			
	PE policy				−0.003 (0.002)	.070	−.25	**−0.003 (0.001)**	.024	−.25
	Transport policy				−0.039 (0.138)	.778	−.06			
Variance components	Individual-level	3.776 (0.057)	< .001		3.776 (0.057)	< .001		3.776 (0.057)	< .001	
	School-level	0.137 (0.013)	< .001		0.137 (0.013)	< .001		0.137 (0.014)	< .001	
	Country-level ^b^	0.106 (0.026)	< .001		0.066 (0.014)	< .001		0.066 (0.015)	< .001	

*Ntl.* National, *PA* Physical Activity, *PE* Physical Education, National income divided by 1000 for interpretability of *b* values

^a^Female is reference group.

^b^Explained country-level variance = (106–.066)/.106 = .38

p<.05 are set in bold

Table 4 displays results of VPA analyses, with models showing that both gender and age were significant individual-level correlates of VPA, with less VPA found among girls and younger adolescents. Model 1 shows significant school- and country-level variance, with a residual ICC of 3.9% at school-level and 4.6% at country-level. Model 2 included all country-level variables, and national temperature, urbanisation, adult physical activity, safety and national income met the criterion for inclusion in the final model. In a model with these five variables, safety was no longer significant (*b* = 0.007, *p* = .073). Model 3 shows that when the remaining four variables were included in a regression together, lower national temperature, greater urbanisation, more adult physical activity, and higher national income were significantly associated with more VPA. 81% of the total country-level variance was explained by these four variables.

**Table 4:** Multilevel models for vigorous-intensity physical activity with unstandardised and standardised fixed effects at individual- and country-level (*N* = 138,014)

		Model 1			Model 2			Model 3		
		*b* (SE)	*p*	*β*	*b* (SE)	*p*	*β*	*b* (SE)	*p*	*β*
Fixed effects (individual -level)	Intercept	1.709 (0.218)	< .001		−2.745 (1.144)	.415		−1.992 (0.961)	0.011	
	Gender ^a^	**0.738 (0.049)**	< .001	.16	**0.738 (0.049)**	< .001	.16	**0.738 (0.049)**	< .001	.16
	Age	**0.039 (0.014)**	.005	.03	**0.039 (0.014)**	.005	.03	**0.039 (0.014)**	0.005	.03
Fixed effects (country-level)	Ntl. temperature				**−0.035 (0.011)**	.002	−.23	**−0.034 (0.010)**	.001	−.23
	Urbanisation				**0.013 (0.005)**	.004	.32	**0.015 (0.004)**	< .001	.36
	Adult PA				**0.028 (0.010)**	.005	.42	**0.023 (0.009)**	.009	.34
	Safety				**0.008 (0.004)**	.037	.17			
	Ntl. income				**0.066 (0.016)**	< .001	.67	**0.064 (0.012)**	< .001	.65
	Income inequality				0.004 (0.013)	.767	.03			
	PE policy				−0.003 (0.002)	.247	−.14			
	Transport policy				−0.115 (0.109)	.288	−.12			
Variance components	Individual-level	4.923 (0.118)	< .001		4.923 (0.118)	< .001		4.923 (0.118)	< .001	
	School-level	0.208 (0.025)	< .001		0.207 (0.025)	< .001		0.207 (0.025)	< .001	
	Country-level ^b^	0.248 (0.058)	< .001		0.038 (0.009)	< .001		0.046 (0.013)	< .001	

*Ntl.* National, *PA* Physical Activity, *PE* Physical Education. National income divided by 1000 for interpretability of *b* values

^a^Female is reference group

^b^Explained country-level variance = (.248–.046)/.248 = .81

p<.05 are set in bold

Sensitivity analyses revealed that the results were robust to changes in the definitions and coding of several variables. The inclusion of an individual-level measure of family socioeconomic status – the Family Affluence Scale (FAS, which was available for all countries except Lithuania and Spain) did not substantially affect the models. An alternative measure of national wealth, a log-transformed measure of GDP, showed an association between higher GDP and more adolescent VPA, but GDP was not associated with MVPA. An alternative measure of annual average national temperature, using World Bank data aggregated across each country (rather than only in major cities), made no substantial difference to results. Likewise, transport policy remained an insignificant predictor when coded as 0 for “no policy”, 1 for “policy stated but only partially implemented or enforced” and 2 for “policy entirely implemented and enforced”.

## Discussion

This study shows that national differences in the physical, socio-cultural, economic and policy environment were associated with individual differences in adolescent physical activity. Characteristics of the national environment explained a large amount of the international variation: 81% of country-level variance in VPA and 38% of country-level variance in MVPA. Adolescents did more MVPA in countries with lower annual average national temperatures, higher perceptions of community safety, lower average national income and weaker physical education policies. More adolescent VPA took place where there was a lower annual average national temperature, a higher percentage of urban areas, more adult physical activity and a higher national income. The findings show that combinations of characteristics from different environment types best explained both MVPA (physical, socio-cultural, economic and policy) and VPA (physical, socio-cultural and economic), and as such indicate the usefulness of the ANGELO framework. Furthermore, different environmental factors were associated with MVPA and VPA, showing a different underlying explanatory pattern for the two behaviours.

Both physical environment measures were associated with physical activity. There was more adolescent MVPA and VPA in countries with lower national temperatures, an association also found for adults [24]. Another study looking at adolescent physical activity across countries also found evidence that, while activity levels are higher during warmer months in many countries, physical activity reduces once mean temperature reaches above 20 °C [29]. Evidence of more VPA (but not MVPA) in more urbanised countries suggests that facilities may be important for vigorous exercise but not so relevant for everyday activity [60], consistent with previous findings (e.g., [61]).

This interpretation of the results for urbanisation, may also support the unexpected findings for national income, with higher national income associated with more VPA, but lower national income associated with more MVPA. The latter result contrasts with analysis of 2002, 2006 and 2010 HBSC data, which found increases in gross national income (GNI) were related to more MVPA [38]. Sensitivity analysis revealed that GDP (which was more closely correlated with GNI than average national household income) was not associated with MVPA. Thus, it seems likely that average national household income, which is considered a better measure of material living standards than GDP [52], captures something significantly different than either GDP or GNI. It is possible that higher national income encourages engagement in organised sport and exercise, contributing to VPA, but may also encourage motor vehicle usage for daily travel while discouraging MVPA (i.e. cycling and walking).

Income inequality was not associated with MVPA or VPA. Our findings suggest that previous evidence of links between income inequality and physical activity could be due to the correlations between income inequality and national temperature and safety (MVPA) and urbanisation (VPA) [38]. At the country-level, higher family affluence has been associated with more physical activity (the finding is stronger for VPA than MVPA [13]), but in this study of country-level factors, controlling for individual-level family affluence did not substantially affect the model.

Concerning the socio-cultural environment, there was more adolescent MVPA and VPA in countries perceived to be safer, although the association between VPA and safety was no longer significant in the final model. Findings that community safety perceptions aggregated at the national level were related to physical activity supports evidence of links between parental perceptions of neighbourhood safety and their children’s physical activity [62]. These findings suggest that MVPA may involve higher levels of independent mobility where safety plays an important role (e.g., [63]), whereas VPA, which is more likely to take place in the context of supervised sport or exercise, is less dependent on perceptions of safety. The national level of adult physical activity was significantly associated with VPA but not with MVPA. Adolescents may benefit from a culture of active adults which encourages young people to be involved in sport and exercise [31, 32]. The lack of findings for MVPA accords with the evidence found in reviews of physical activity correlates which show inconsistent evidence of older family members’ influence on individual physical activity [20, 27].

Countries with strong physical education policies (i.e., a greater required minimum annual taught time for physical education) were unexpectedly likely to have lower adolescent MVPA. Strong policies may be a legislative response to low levels of physical activity and may be in the process of implementation. Alternatively, implementation of policies may be suboptimal, with actual practice deviating from official policy [7]. The lack of an effect of physical education policy on VPA may be due to the measure of VPA, which measures exercise outside school hours. The impact of transport policy may not be effectively evaluated using a measure of single policy item. A combination of physical activity-enhancing policies in urban planning, transport, infrastructure and education domains may be necessary to create an environment for more physical activity [7]. Even in just one domain an accumulation of policies and facilities can have a greater effect on physical activity than policy alone [64]. Measures that capture the broader policy agenda, such as a ‘global matrix’ of internationally comparable indicators of the physical activity environment in adolescence, may be useful for further analyses [12]. Transport policies at the national level may be quite independent from those at the regional or even school level, which may be more influential [65].

### Strengths and limitations

Some limitations of the study should be considered. Firstly, causal relationships between the environment and physical activity cannot be confirmed using such cross-sectional research. However, individual physical activity is unlikely to explain environmental factors such as national temperature, urbanisation or national income. Either these environmental factors have a causal effect or there are confounders which cause both outcome and predictor. Combining these findings with those of longitudinal and experimental research would enable researchers to come to firmer conclusions about causality [66, 67]. Secondly, the results may not be generalisable beyond higher-income European countries. Effects of country-level factors could be even stronger with a more diverse sample of countries, although environmental impacts on physical activity differ between higher- and lower-income countries [20, 46]. Thirdly, with data from only 29 countries included, the final models should be interpreted with caution. Significant correlations between country-level determinants might have left the study with insufficient power to determine associations. However, without high-quality, contemporaneous, comparable data for many countries it is difficult to study many national environmental effects [68].

Fourthly, assumptions about country-level indicators may have affected the stability of the results, though these assumptions were necessary to include a reasonable sample of countries. The measurement of safety was carried out in different countries in different years. The measure of income inequality could have included some uncertainty, given the poor comparability of international data [69]. The measurement of transport policy was reliant on the judgement of individual experts and was a categorical dummy variable, which may have limited the power to detect effects from this indicator. Future studies should consider including explanatory variables at the region- or school-level (including temperature, assessments of community safety and policy measures), because national indicators may underestimate the effect of local environmental influences. Measures of annual average temperature also do not capture whether there are substantial or small seasonal effects that cause temperatures to fluctuate throughout the year, which may affect adolescent physical activity [28]. The data in this study were collected at different times of the year in different countries and therefore may be subject to some seasonal effects. Finally, the reliability of self-reported physical activity data can depend on cultural and socio-demographic factors [70]. Further research using different measures would add to the evidence base.

This study’s unique strength is its evaluation of models with multiple national predictors and comparable individual-level data from 29 countries. The results suggest that researchers studying country-level influences on physical activity should consider including different types of environmental factors into their models and avoid assumptions that physical, economic, socio-cultural or political factors alone explain international differences in MVPA and VPA. Inconsistent findings regarding environmental correlates (see [26, 31]) may be partly explained by the presence (or absence) of other relevant environmental covariates in researchers’ models.

## Conclusion

Despite considerable cross-national variability in adolescent physical activity, explanations of this variability are scarce. Previous research examining the role of environmental factors has given limited attention to the relative importance of different types of environmental factors, so this study examined eight different factors in a single design. The findings that physical, socio-cultural, economic and political environment factors are all related to physical activity provide support for ecological theory and emphasise the importance of taking different environmental factors into account simultaneously. Future research should acknowledge the conceptual difference between MVPA and VPA, given they are associated with different environmental factors. Intervention planning must therefore consider how environmental strategies affect both moderate and vigorous physical activity. Notwithstanding the effects we found on the country-level, effects on the individual level – where most of the variation in adolescent physical activity takes place – are much stronger. However, from a scientific and public health perspective it is important to know how the country-level variance can be explained. Further studies could examine mechanisms through which country-level factors affect adolescent physical activity or look more closely at interactions between individual and environmental factors, which might shed light on cross- national gender, age and socio-economic differences [16]. This may help increase physical activity in groups where it is low, such as girls. With low levels of physical activity in adolescents worldwide, it is vital that researchers continue to investigate the determinants of both daily physical activity and vigorous exercise.

## Additional file

Additional file 1: Information on ethical approvals in the HBSC study 2009/2010, provided by the principal investigator of each country. (DOCX 16 kb)

## Abbreviations

ANGELO: Analysis Grid for Environments Linked to Obesity
EU-SILC: European Union Statistics on Income and Living Conditions
FAS: Family Affluence Scale
GDP: Gross domestic product per capita
GNI: Gross National Income
HBSC: Health Behaviour in School-aged Children
HDI: Human Development Index
ICC: Intraclass correlation
MVPA: Moderate- to vigorous-intensity physical activity
VPA: Vigorous-intensity physical activity
WHO: World Health Organization
