# Supplementary material for: Do country-level environmental factors explain cross-national variation in adolescent physical activity? A multilevel study in 29 European countries
Source: BMC Public Health. 2019 Jun 3;19:680. doi: 10.1186/s12889-019-6908-9 (PMC6547567; doi:10.1186/s12889-019-6908-9)
Supplement: Supplementary file 1 — Information on ethical approvals in the HBSC study 2009/2010, provided by the principal investigator of each country. (DOCX 16 kb) [file 12889_2019_6908_MOESM1_ESM.docx]

**Additional file 1**

Information on ethical approvals in the HBSC study 2009/2010, provided by the principal investigator of each country.

| Country (n=29) | Approval? | Ethics approval information | Principal investigator |
| --- | --- | --- | --- |
| Austria | n/a | - | Wolfgang Dür |
| Belgium (Flemish) | yes | Ethics review committee of the University Hospital of Ghent | Carine Vereecken |
| Belgium (French) | yes | Education Boards of the different School Networks in the Federation Wallonia-Brussels | Danielle Piette |
| Bulgaria | n/a |  | Lidiya Vasileva |
| Croatia | n/a |  | Marina Kuzman |
| Czech Republic | not required | - | Michal Kalman |
| Denmark | not required | - | Pernille Due |
| England (UK) | yes | Ethics Committee of University of Hertfordshire | Fiona Brooks, Antony Morgan |
| Estonia | yes | Tallinn Medical Research Ethics Committee, Tallinn, Estonia | Katrin Aasvee |
| Finland | yes | Trade Union on Education in Finland and the National Board of Education | Jorma Tynjälä |
| France | n/a | - | Emmanuelle Godeau |
| Germany | yes | Ethics Committee of the Medical Association of Hamburg (on behalf of the University Clinic Hamburg) | Petra Kolip |
| Greece | yes | Ministry of Education  Pedagogical Institute | Anna Kokkevi |
| Hungary | yes | Scientific and Research Ethical Committee of the Medical Research Council of the Hungarian Ministry of Health | Ágnes Németh |
| Ireland | yes | Human Research Ethics Committee of the National University of Ireland, Galway | Saoirse Nic Gabhainn |
| Italy | yes | Ethical Board of the Istituto Superiore di Sanità, Roma | Franco Cavallo |
| Latvia | not required | - | Iveta Pudule |
| Lithuania | yes | Kaunas Regional Biomedical Research Ethics Committee | Apolinaras Zaborskis |
| Netherlands | yes | Faculty Ethics Assessment Committee, Faculty of Social Sciences, Utrecht University | Gonneke Stevens |
| Norway | n/a | - | Oddrun Samdal |
| Poland | yes | Ethics Committee at the National Research Institute of Mother and Child, Warsaw | Joanna Mazur |
| Portugal | yes | Hospital S Joao, O Porto University, Ethical committee | Margarida Gaspar de Matos |
| Romania | n/a | - | Adriana Baban |
| Scotland (UK) | yes | School of Medicine Research Ethics Committee, University of St Andrews | Candace Currie |
| Slovakia | yes | Ethics Committee of the Medical Faculty at the P. J. Safarik University in Kosice | Andrea Geckova |
| Slovenia | yes | Republic of Slovenia National Medical Ethics Committee | Helena Jericek |
| Spain | n/a | - | Carmen Moreno Rodriguez |
| Sweden | not required | - | Lilly Augustine |
| Wales (UK) | yes | Ethics Committee of Cardiff University, School of Social Sciences | Chris Roberts |

n/a = not available
